# New York Academy of Medicine

**Published:** 1878-04

**Authors:** 


### NEW YORK ACADEMY OF MEDICINE.

Stated Meeting, February 18, 1878.

Salvatore Caro, M.D., Chairman, read a brief paper upon vicarious menstruation, and gave the history of a case in which the hemorrhage occurred from the gums. The patient was 23 years of age; began to menstruate at the age of 16; had an attack of intermittent fever in September; menses disappeared, but did not return when the malarial fever was arrested. She soon began to suffer from toothache, which was accompanied by some hemorrhage from the gums. Relief not being obtained, in July following she had the tooth extracted at the time it was giving her great discomfort; blood came freely from the cavity, and the bleeding resisted all means, such as plugging, etc., for its arrest, but finally ceased. In August bleeding from the gum returned; application was made to a dentist for relief; none was obtained, but the bleeding again ceased at the end of four days. In September the bleeding from the gum returned; she applied to a physician; no special relief was obtained, but hemorrhage again ceased at the expiration of four days. In October the patient came under Dr. Caro's care. It was regarded as a case of vicarious menstruation, and the patient was placed under the following plan of treatment:

She belonged to a religious order that imposed protracted and severe religious duties; it was advised to abstain from all these; to take plenty of good food; to wash the mouth daily, for about ten days prior to the time for the occurrence of the hemorrhage from the gum, with tincture of myrrh and water; to take thirty drops of the fluid extract of ergot every four hours; and to apply mustard drafts to the popliteal region.

In October she bled a little from the gum and soiled one napkin. The treatment was repeated in November, but not with as favorable results, for no blood was discharged from the uterus, and the bleeding from the gum was more profuse than formerly.

In December the mouth-wash and mustard revulsives were repeated, and ten-drop doses of the tincture of iodine given in Spanish saffron tea; menstruation returned, without hemorrhage from the mouth, and lasted five days. In January the patient menstruated normally; duration, four days. In February the menstrual function was performed in a normal manner; her health seemed perfect; duration of discharge four days.

Dr. Isaac E. Taylor remarked that Dr. Caro's case was important as illustrating the effect produced in the female economy by malaria. Malaria exerted a powerful influence not only with regard to amenorrhœa, but also with regard to metrorrhagia, affections of the kidneys, stomach, intestines, etc., etc. Hence, the disturbances of the nervous system and the development of neuralgia, toothache, gastralgia, etc. The effect produced upon the system by malaria during pregnancy was much more marked than under other circumstances. Within the past year he had seen six cases of gastralgia dependent upon malaria, and all occurred in pregnant women. Now, when menstruation ceased because of the action of malaria in the system, as it seemed to in the case reported by Dr. Caro, hemorrhage might occur at any point where the blood-vessels were in a weakened condition, whether it was in the mouth, upon the arm, or upon the top of the head. Dr. Taylor referred to one case in which the vicarious hemorrhage occurred from a patch upon the arm; also to another in which it occurred from a broken surface upon the top of the head. Mention was also made of a case in which hæmatemesis was substituted for menstruation. The uterus was measured and found to be 3½ inches in depth. A sound, having a large end, was introduced a few times prior to the time for the occurrence of menstruation; the uterus was increased in size to a little over four inches, owing to the normal ductility of the organ; menstruation came on, and then the uterus was very much reduced in size; a process of involution, as it were, was accomplished. At the end of two months the menstrual flow was fully established. If that plan of treatment was adopted, the sound must be used with the greatest gentleness and care.

Dr. Hubbard referred to a case in which the patient was supposed by her physicians to have hemorrhage from the lungs, because at each menstrual period she expectorated blood. The expectoration of the sanguineous fluid lasted three or four days; she was then entirely free from it until the next menstrual period, and the phenomenon continued until near the close of her menstrual life. The woman was in very good health, and no treatment was introduced except that directed to her hysteria.

Dr. Church mentioned a case in which hemorrhage occurred monthly from the umbilicus; but the history was incomplete, from the fact that circumstances were such as to forbid complete examination. There was no menstrual discharge per vaginum, and the Doctor was unable to say whether or not a vagina existed.

Reference was made by several members to cases in which existing discharges, such as discharge from the ears, from ulcers, etc., etc., were aggravated at the menstrual period.

*Placenta Prævia without Hemorrhage.*—Dr. Isaac E. Taylor related the history of a case in which the placenta presented, yet delivery was accomplished without hemorrhage and almost without pain.

The woman was æt. 25, not very robust, and at nearly full term presented an appearance as if only in the fifth or sixth month of pregnancy. The Doctor was called to decide whether or not the child was living. Neither placental murmur nor fœtal heart could be heard. By external manipulation the conclusion was reached that the head of the child was in the cavity of the pelvis, thus accounting for the apparently small size of the child. The Doctor called again at about four o'clock, when, on examination, the neck of the uterus was found to be about one inch in length, and undilated; pains very slight and irregular; and the finger came in contact with a globular mass, which was quite soft as felt through the uterine wall.

At 6½ P.M. Dr. Taylor was summoned, and found the pains quite regular, the neck dilatable, so as to permit the introduction of a finger, and a very soft substance was felt; the ordinary show was absent; there was quite an abundant secretion of mucus; and he was unable to determine the nature of the soft mass.

At about 7.20 P.M., with a single expulsive effort, the placenta came down, child and all, and the labor was completed without the loss of blood. On examination the head of the child was found to be hydrocephalic; the placenta was 3½ inches in width and in a complete state of fatty degeneration.

Dr. O'Sullivan mentioned a case in which there was placenta prævia, but no hemorrhage, and when labor was completed the placenta was found to be the seat of complete fatty degeneration.

The section then adjourned to the dining-hall, where the members and guests, on invitation by the Chairman, partook of a bountiful collation.—*N. Y. Medical Record.*

